# Anterior Ischemic Optic Neuropathy as a Manifestation of HELLP Syndrome

**DOI:** 10.1155/2014/671976

**Published:** 2014-09-29

**Authors:** Boby Varkey Maramattom

**Affiliations:** Department of Neurology, Aster Medcity, Kochi, Kerala 682023, India

## Abstract

Thrombotic microangiopathies (TMAs) are a group of disorders characterized by occurrence of thrombi of fibrin and/or platelets with microvascular occlusion and organ ischemia especially the kidney and brain. Hemolysis with a microangiopathic blood smear, elevated liver enzymes, and low platelet count (HELLP syndrome) is a type of TMA peculiar to pregnancy and may be associated with neurological complications. Visual complications in HELLP are usually related to cortical blindness. We present the first case of HELLP associated with bilateral anterior ischemic optic neuropathy (AION) and blindness which resolved with plasma exchange.

## 1. Introduction

Thrombotic microangiopathies (TMAs) are a group of disorders characterized by occurrence of thrombi of fibrin and/or platelets in the microvasculature of various organs, mainly the kidney and brain, due to complement dysregulation, ADAMTS-13 deficiency, or Verotoxin and VEGF deficiency [1]. Hemolysis with a microangiopathic blood smear, elevated liver enzymes, and low platelet count (HELLP syndrome) is a rare TMA peculiar to pregnancy. Neurological complications occur in a large proportion of patients, but visual loss is usually due to cortical blindness [2]. Although bilateral AION has been described in one case of severe preeclampsia, there are minor but significant differences between preeclampsia and HELLP syndrome. We present the first case of HELLP syndrome with reversible blindness caused by bilateral anterior ischemic optic neuropathy (AION) and we will review the difference between HELLP syndrome and severe preeclampsia [3].

## 2. Case Report

**Figure 1:**
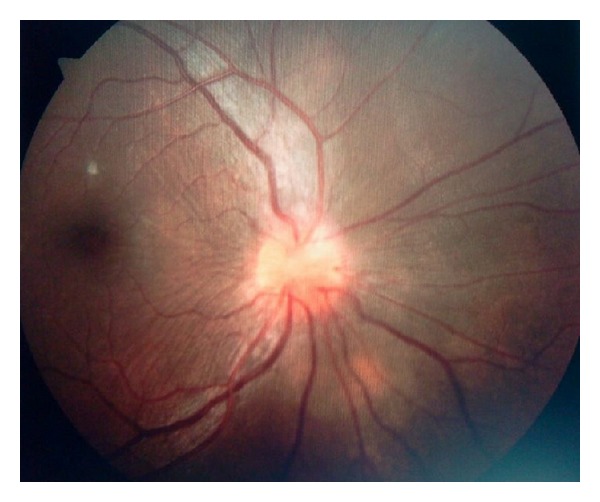
Fundus picture showing a congested right optic disc suggestive of AION.

**Figure 2:**
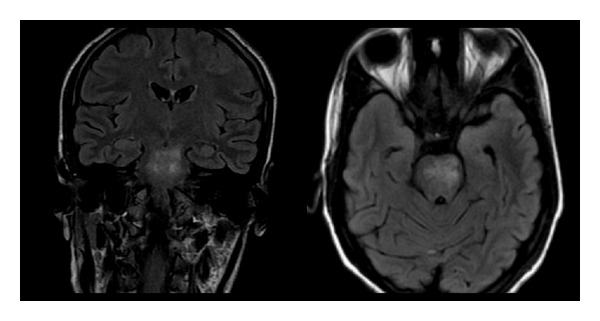
Coronal and axial FLAIR images showing pontomesencephalic hyperintensities suggestive of PRES syndrome.

**Figure 3:**
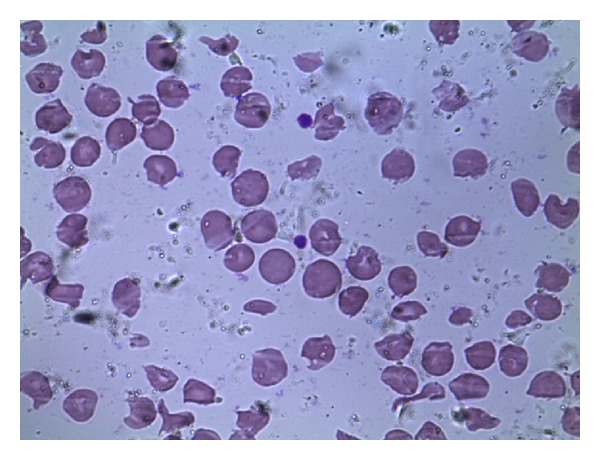
Peripheral smear showing polychromasia, helmet cells, fragmented RBCs, and anisopoikilocytosis.

A 23-year-old primigravida presented at 33 weeks complaining of painless visual loss in her right eye since morning. On examination, she had a BP of 150/100 mm Hg, urine proteinuria 2+, visual acuity of finger counting at 4 meters in the right eye, and a Marcus Gunn pupil and fundoscopy showed a congested right optic disc (Figure 1). Visual evoked potentials (VEP) showed a prolonged P100 latency at 120 msecs. She was taken up for elective LSCS the next morning. On day 3, her vision in the right eye had declined to light perception. IV methylprednisolone was started at a dose of 1 gm per day for 5 days. Her platelet count was 100,000 per mm^3^. The next day, her platelet count was 75,000, LDH was 675, total bilirubin was 1.3 mg/dL, AST was 65, ALT was 57 U/L, and LDH; AST ratio was 10.4. RBC indices and peripheral smears were normal. On day 5 contrast MRI of the brain was suggestive of brainstem posterior reversible encephalopathy syndrome (PRES) (Figure 2). On day 6 she developed painless total visual loss in the left eye. Fundoscopy showed left optic disc congestion with a single peripapillary hemorrhage. Repeat VEP study showed prolonged P100 in both eyes, right (125 msec) and left (120 msec), with decreased amplitudes in the left eye. The diagnosis was revised to bilateral anterior ischemic optic neuropathy (AION) and PRES. She was started on IV antihypertensives and Fosphenytoin for seizure prophylaxis. By evening, she became drowsy and became comatose overnight. Both pupils were dilated and unreactive to light. A repeat MRI brain showed no new changes. Platelet count was 45000 per mm^3^ and peripheral smear now showed features of microangiopathic hemolytic anemia (Figure 3). ANA, RA factor, APLA antibodies, lupus anticoagulant, anticardiolipin antibodies, ANCA, VDRL, HBsAg, and ELISA for HIV were negative. At this point of time, a TMA (HELLP syndrome) was considered and she was taken up for plasma exchange (PLEX) due to postpartum worsening. After 2 cycles of PLEX, her sensorium had improved. After 6 cycles of PLEX, her pupillary reflexes reappeared and platelet counts normalized. At followup 6 months later, she had a visual acuity of 20/30 bilaterally.

## 3. Discussion

Thrombocytopenia (platelet counts < 150,000 cmm^3^) is seen in about 10% of pregnancies [4, 5]. Most cases are benign and usually only the TMAs are associated with neurological complications (Table 1). HELLP syndrome is a TMA peculiar to pregnancy often occurring before the 37th week of pregnancy, accompanied by significant morbidity and mortality if diagnosed at a late stage. Patients often have abdominal pain and vomiting and neurological complications occur in >50% of patients with HELLP syndrome in some series, either seizures, focal neurological deficits, or encephalopathy [2, 6].

Although patients with HELLP syndrome and preeclampsia look similar, the major difference is the involvement of the coagulation system in HELLP patients unlike preeclampsia. Moreover, the histopathologic profile and the types of placental lesions differ between HELLP and PE placentas, suggesting different pathogenetic mechanisms. Thereby a significant number of small for gestational age (SGA) babies are born in preeclamptic mothers as compared to HELLP. Therefore it is important to differentiate between the two disorders. Neuroimaging usually shows features of posterior reversible encephalopathy (PRES), although intracranial hemorrhage or subarachnoid hemorrhage may be seen. 10%–20% of women with HELLP syndrome have severe preeclampsia but 20% do not have antecedent hypertension or proteinuria. Criteria for the diagnosis of HELLP syndrome include hemolysis (abnormal peripheral smear, LDH > 600 U/L, or bilirubin > 1.2 mg/dL), AST > 70 U/L, and a platelet count < 100,000/cmm^3^ [7]. The University of Mississippi criteria are helpful in categorizing the severity of HELLP syndrome and are predictive of the maternal mortality rate [8]. The clinical features of various TMAs can sometimes be extremely difficult to differentiate especially between HELLP syndrome and TTP. As in our patient, an LDH to AST ratio <22.12 may be more in favor of HELLP syndrome than TTP in the third trimester pregnant patient [9]. The treatment also differs slightly in that TTP requires PLEX, methylprednisolone, or rituximab whereas HELLP usually resolves after delivery. Rarely, postpartum worsening of HELLP may respond to PLEX, as in our patient [10].

Visual abnormalities in the context of HELLP syndrome usually involve cortical blindness, retinal detachment, or retinal hemorrhages [11, 12]. To our knowledge, this is the first case report of AION associated with HELLP syndrome.

## Figures and Tables

**Table 1:** Thrombotic microangiopathies in pregnancy.

Specific to pregnancy	HELLP syndrome Acute fatty liver of pregnancy [1]

Not specific to pregnancy	TTP/HUS SLE Antiphospholipid antibody syndrome [APLAS] CAPLAS DIC

CAPLAS: catastrophic antiphospholipid antibody syndrome, SLE: systemic lupus erythematosus.
